# Validation of an Image Analysis Method for Evaluating the Chemical Resistance of Glass Fibers to Alkaline Environments

**DOI:** 10.3390/ma15010161

**Published:** 2021-12-27

**Authors:** Martina Ryvolová, Lucie Svobodová, Totka Bakalova

**Affiliations:** Department of Material Science, Faculty of Mechanical Engineering, Technical University of Liberec, 461 17 Liberec, Czech Republic; Martina.Ryvolova@tul.cz (M.R.); Totka.Bakalova@tul.cz (T.B.)

**Keywords:** glass fiber, alkaline environment, image analysis, corrosion process, fiber weight loss, fiber tensile strength

## Abstract

This article is focused on the comparison of the reliability of the results obtained by image analysis (newly proposed evaluation method) with well-known methods of evaluation of long-term corrosion resistance of glass fibers in an alkaline environment (pH > 12). The developed method is based on the analysis of scanning electron microscopy images (diameter and structures on the fiber surface). An experiment (52 weeks) was performed to evaluate two types of glass fibers: anticorrosive glass fibers (ARGFs) and E-glass fibers (EGFs). Three media were used to treat the fibers (23 ± 2 °C): H_2_O, Ca(OH)_2_, and K_2_SiO_3_. The ARGFs’ tensile strength did not reduce; a decrease by 68% was observed for EGFs in H_2_O. Tensile strength decreased by 32% and 85–95% in K_2_SiO_3_; by 50% and 64% in Ca(OH)_2_ for the ARGF and EGF, respectively. Statistical analysis was performed to validate the reliability and plausibility of the developed method. ARGFs and EGFs did not show any relationship between the fiber diameter and weight in H_2_O; however, the linear trends may predict this relationship in Ca(OH)_2_ and K_2_SiO_3_. For the ARGF and EGF, the cubic trend was suitable for predicting the change in fiber weight and diameter over time in Ca(OH)_2_ and K_2_SiO_3_.

## 1. Introduction

Mineral fibers include a range of glass and basalt fibers, and they have good mechanical properties such as high tensile strength, high tensile modulus, non-flammability, dimensional stability, and good resistance to heat, fungi, and microorganisms. Thus, they are commonly used as reinforcement or dispersed in composites where the matrix material has low tensile strength, such as polymers, ceramics, concrete, and aluminosilicate mixtures. Fiber reinforcement increases the stiffness, compressive and flexural strengths, and toughness of the composite, and it can affect the thermal, electrical, and other properties [1]. Mineral fibers include a range of glass fibers and basalt fibers which also have different oxide representations (e.g., Ca, Mg, Fe, Al, K, Na, B), low resistance, and instability in an alkaline environment [2,3] such as concrete (pH = 12–13) or geopolymer matrices (pH = 12–14). The fibers are damaged by chemical reactions that take place in the matrix, which is characterized by the breaking of Si–O–Si bonds within the fibers [1]. The chemical resistance of glass and basalt fibers varies according to the fiber composition (elements/oxides), temperature, solution concentration, and exposure time.

E-glass fibers (EGFs) are made of alkali-free glass (<1% alkali) and have poor chemical resistance to acids and alkalis [3]. They are used to reinforce polymer matrices. EGFs typically contain B_2_O_3_ and CaO, in contrast to basalt fibers that contain FeO and Fe_2_O_3_. Both EGFs and basalt fibers contain SiO and Al_2_O_3_ [4]. AR glass fibers (ARGFs) contain ZrO_2_ and are boron-free. Their composition provides the necessary alkali resistance in cement mortars and concrete [5]. The addition of 12–16 mol% ZrO_2_ significantly improves the resistance of fibers to alkaline environments and suppresses corrosion [1]. However, increasing the ZrO_2_ content worsens melt spinning, and the optimal content has been determined to be 11 mol% [6]. Mineral fibers corrode according to two modes. In the first mode, a brittle shell is created on the fiber surface. The water molecules in the alkaline solution cause the shell to swell, which reduces its cohesion and causes it to crack and peel. The fiber underneath the shell remains intact, and the fiber diameter decreases. The whole cycle is repeated over time [4,7,8,9,10,11]. In the second mode, small holes form on the fiber surface, which increases in size to significant holes and cracks. The small cracks gradually merge, which leads to considerable surface damage. However, the fiber diameter does not change [7,10,12].

In the construction industry, glass fibers are expected to have a service life of several decades. The service life of the fibers can be increased by using low-alkalinity cement, which is also called sulfoaluminate cement. Another approach to reducing the rate of degradation is to create a barrier between the fibers and matrix. Glass fibers are primarily protected by a thin layer (approximately 20 nm thick) of organosilane reagent (i.e., sizing), which allows fibers to adhere to the matrix by creating an interface [2]. Sizing also reduces the number and size of cracks on the fiber surface. Defects that occur on the fiber surface during the manufacturing process serve as stress concentrators that affect the service life and cause damage under load [13]. Another approach to protecting fibers is to coat them with PVA-type polymers, which prevents the diffusion of cement, and is called glass fiber reinforced polymer (GFRP) [14]. The polymer on the fiber surface protects against aggressive environments and ensures better matrix adhesion to the fibers as well as resistance to abrasion, moisture, or temperature [15]. GFRPs are commercially available under the trade name CemFil [1]. The alkaline resistance of glass fibers can be ensured by adding about 1% ThO_2_ and 6% ZrO_2_ to the glass melt. Due to its radioactivity, the thorium content should not exceed 4% [16]. These materials increase the fiber resistance by developing a coating based on a self-crosslinking butadiene–styrene polymer. The thickness of the coating affects the diameter and overall strength of the fibers in an alkaline environment [17]. Many works have considered the behavior of mineral fibers in cement or geopolymer mixtures. The corrosion of the fibers themselves affects the behavior of the composite and its properties depending on the environment, temperature, and exposure time [18,19,20].

Chemical resistance tests are generally based on determining the content of alkali ions in the leachate. Standards such as ASTM C 1203-91 and ISO 695 have implemented accelerated corrosion tests that use a solution with a high pH and elevated temperature to create highly aggressive conditions. The loss of fiber weight can be used to determine the rate of corrosion due to the environment [7]. However, the long-term chemical resistance of mineral fibers cannot be accurately determined by using one or two methods. The fiber tensile strength test is another method for determining the chemical resistance [4,8,10,11]. The tensile strength of corroded basalt fibers shows two trends: a general decrease with temperature or exposure time, a cyclical decrease, and an increase depending on the phase of corrosion shell formation [4,7,10]. Scanning electron microscopy (SEM) has been used to monitor and describe changes in fiber surfaces [4,7,8,9,10,11,17]. Fȍrster et al. and Ying et al. investigated the growth of corrosion shells on the surface of basalt fibers with different compositions [12,21]. They embedded untreated and alkaline-treated fibers in epoxy resin and prepared perpendicular cuts. In the initial phase, they observed that the fiber diameter increased, followed by the formation of a shell enriched with Si, Na, and Al. Their analysis of the reaction kinetics confirmed that the resistance of the basalt fibers to the alkaline environment improved with a higher MgO content [21].

A new method was developed that uses image analysis to monitor the corrosion rate of glass fibers. In this study, the performance of the developed method was compared against that of conventional methods. Statistical analysis was performed to confirm the significance of the identified trends, patterns, and relationships, which have not previously been considered in this field (to the best of our knowledge). This study is significant because the proposed method facilitates non-destructive testing, and the results establish correlations between the image analysis of the proposed method and indicators commonly used by conventional methods (weight change, fiber tensile strength).

## 2. Materials and Methods

### 2.1. Fibers and Pretreatment Process

In this study, rovings (StarRov) of E-glass fibers (EGF) from Johns Manville Company (Trnava, Slovakia) were used containing filaments with a diameter of 16 µm and nominal weight of 2400 tex. Rovings of ARGFs (commercial name: Cem-Fil, type Anti-Crak^®^ HD, Sklocement Beneš s. r. o. Company, Ostrava, Czech Republic) from Owens Corning were used with a nominal weight of 2400 tex and filament diameter of 12 µm. The ARGFs were doped with ~9 wt. % ZrO_2_, which the manufacturer claimed gave them excellent resistance to alkaline environments. The fibers were treated in the following concentrated alkaline solutions: distilled water saturated with calcium hydroxide (Ca(OH)_2_, manufacturer Sigma Aldrich, Prague, Czech Republic) at pH 12, concentrated potassium water glass (K_2_SiO_3_, manufacturer ČLUZ a. s., CR) at pH 14, and distilled water at pH 7. The fibers were immersed in the solutions at room temperature (23 ± 2 °C) for 1 year (52 weeks) and were monitored at 2, 4, 6, 13, 26, and 52 weeks (represented as W2–W52).

### 2.2. Fiber Resistance and Static Tensile Strength Test

The resistance of the glass fibers to an alkaline environment was measured according to the standard ISO 695 [22]. Samples were divided into two groups: untreated (i.e., Ref. as reference samples) and solution-treated. Samples were prepared according to the standard ISO 11566 [23]. The brittle glass fibers were inserted into a paper frame to protect them from compression by the pneumatic jaws. The static strength test was carried out to determine the changes in the mechanical properties of the treated and untreated fibers. The tensile strength test was performed by using a Testometric device according to EN ISO 13934 [24] to measure the ultimate load. The test conditions were kept stable with a room temperature of 23 ± 2 °C and relative humidity of 50 ± 3%. The deformation velocity was set at 10 mm/s, and the breaking length was set at 100 mm.

### 2.3. Fiber Weight and Maximum Breaking Force

The weight of treated glass fibers was measured by using an analytical balance (Mettler Toledo XSR 105, Prague, Czech Republic). The measured weight and maximum force measured during the static strength test were used as initial values to calculate the tensile strength σ (MPa):

(1) $$\sigma =\frac{F_{max}}{T}\rho_{GF}$$

where *F_max_* is the maximum breaking force (N), *T* is the nominal weight (tex), and $\rho_{GF}$ is the density of the glass fibers (g/cm^3^).

### 2.4. Calculation of the Theoretical Chemical Resistance

The theoretical chemical resistance *R_z_* [25] of the treated glass fibers was calculated according to Equation (2), and the relative rate of degradation *K_p_* was calculated according to Equation (3):

(2) $$R_{z}=\frac{M_{k}}{M_{p}}\;100\%$$

(3) $$K_{p}=\frac{M_{p}-M_{k}}{t_{p}M_{p}}=\frac{100-R_{z}}{t_{p}}$$

where *R_z_* is the chemical resistance (%), *M_k_* is the nominal weight (g), $M_{p}$ is the weight of the treated glass fibers (g), *K_p_* is the relative rate of degradation (%/h), and *t_p_* is the exposure time (h).

### 2.5. Fiber Surface Observation Using SEM and Image Analysis

Scanning electron microscopy (UHR FE-SEM Carl Zeiss ULTRA Plus device, Potsdam, Germany) with an electron high tension of 5 kV, magnification of 2500×, a scan speed of 6, and a working distance of 5.9 mm was used to observe the changes to the fiber surface during the corrosion process. The conductivity of the glass fiber samples was ensured by sputtering a thin layer of platinum using an ion-dusting system SEM Mill model 1060 (producer Fischione Instruments, Inc., Export, PA, USA) and a combined sprayer/steamer Quorum Q15R ES. Image analysis was performed in MATLAB (The MathWorks, Inc., R2019a, Natick, MA, USA). For the proposed method, the original images were preprocessed by histogram balance, noise reduction, and gamma correction. Sharp borders were detected to identify fibers in the images (edge detectors). Both edges and localized points of interest (the fiber surface with corrosion debris) were detected. Then, orientation parameters (the length, object width, and fiber diameter) were classified for the detected objects. The most crucial parameter was the diameter of an elementary fiber (one fibril within a fiber roving). The above image analysis method has also been described in our previous studies [26,27].

### 2.6. Statistical Analysis

Statistical analysis was performed using the external program Statistica version 12 (StatSoft, Inc., CA, USA, www.statsoft.com, 2013, accessed on 11 December 2021). The measured values (weight, fiber diameter, and ultimate load) were processed into box-plot graphs to compare the frequency distributions of datasets and to identify or eliminate outliers. Box-plots are used in descriptive statistics to visualize numerical data as the quartiles Q_25_, Q_50_, and Q_75_ or Q1, Q2, and Q3. A box-plot requires determining and calculating the following characteristics and values: the quartiles *x*_25_, *x*_50_, and *x*_75_; quartile range; ends of rays (A and B). Data in bar charts were reported as the mean ± standard deviation. The Student’s *t*-test was performed to analyze the significance of data (i.e., differences between groups) when appropriate. Data were considered significant when the *p*-value was less than 0.05. Scatter plots (weight versus time, fiber diameter versus time, and weight versus fiber diameter) for individual solutions (H_2_O, Ca(OH)_2_, K_2_SiO_3_) were plotted together with regression bands at the confidence level of 0.95, and regression equations were calculated for linear, quadratic, and cubic dependencies. The dependencies of the three variables (time, weight, and fiber diameter) were graphed as 3D wafer plots. The other parameters were used by Statistica automatically.

## 3. Results and Discussion

### 3.1. Tensile Strength

**Figure 1:**
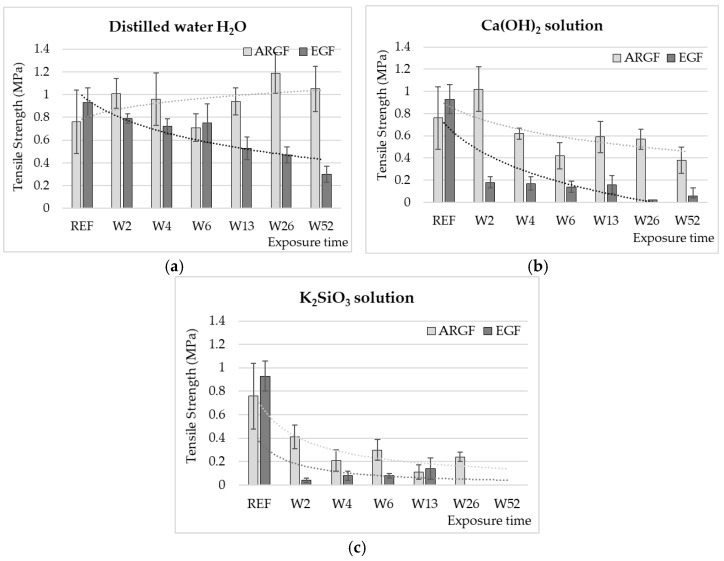
Strengths of ARGFs and EGFs (average value of 10 measurements at minimum, excluding outliers) after treatment in a neutral environment: (**a**) H_2_O and alkaline environments, (**b**) Ca(OH)_2_, and (**c**) K_2_SiO_3_ solutions depending on exposure time (x-axis, in weeks). The dotted lines in the graphs indicate the trend lines.

Figure 1 presents the changes in the tensile strength of the glass fibers (ARGF, EGF) in different environments (H_2_O, Ca(OH)_2_, K_2_SiO_3_) over time (W2–W52). In the case of H_2_O (Figure 1a), the tensile strength of the ARGFs increased by 32% compared with the reference sample after W2. After W6, the tensile strength decreased by 8%, after W52 increased by 38% compared with the reference sample. For the EGFs, the average tensile strength gradually decreased in the period W2–W52. After W52, the average tensile strength decreased by 68% compared with the reference sample (Figure 1a).

In the case of Ca(OH)_2_ (Figure 1b), the tensile strength clearly decreased with increasing exposure time for both types of glass fibers. For the ARGFs, the tensile strength increased by 25% in W2 compared with the reference sample. The tensile strength decreased by 18% in W4 and 44% in W6. This decrease is statistically significant, with a confidence level of *p* < 0.05. At W52, the tensile strength decreased by 50%; all results were compared with the reference sample. For the EGFs, the tensile strength decreased significantly by 80% in W2. In the following weeks (W4 to W13), the tensile strength values of the E fibers were maintained in the range of 0.14 to 0.18 MPa. At the end of the experiment (W26 to W52), the decrease in tensile strength of EGFs in the range from 93 to 97% was evaluated, and the standard deviation of the measurement was significant, with a confidence level of *p* < 0.05. The fibers were very brittle at this time. The slight increase in strength at W26–W52 was probably caused by the growth of mineral incrustations on the fiber surface [18]. During the experiment, the fibers were observed to change in color. The fibers were initially glossy and then changed to milky white over time. 

In the case of K_2_SiO_3_ (Figure 1c), the tensile strength of the glass fibers (ARGFs and EGFs) significantly decreased at the beginning of the experiment. For ARGFs, the tensile strength decreased by 45% (W2) and by 60 to 70% (W4–W6) compared with the reference sample. In the following weeks (W13 to W26), a decrease in the tensile strength of ARGFs of up to 85% compared to the reference value (0.76 MPa) was measured. The variation in the tensile strength (Figure 1c, ARGFs) corresponded to the behavior described in [7], where the insoluble incrustations created on the fiber surface (i.e., corrosion shell) [11] temporarily increased the strength by healing microcracks. The K_2_SiO_3_ solution damaged the fibers to the point that it was impossible to perform a tensile test after 52 weeks of exposure. For the EGFs, the tensile strength decreased by 85–95% compared with the reference value in period W2–W13. The EGFs were dissolved in K_2_SiO_3_ entirely by W26.

These results indicate that long-term exposure to H_2_O at room temperature did not reduce the tensile strength of the ARGFs but did reduce the tensile strength of the EGFs. A reduction in tensile strength of 68% was measured. This can be attributed to the slow-growing Si layer on the EGF surface [12], which reduced the mechanical strength. Gang Wu et al. performed a similar experiment by immersing EGFs in H_2_O at 55 °C for 7, 18, 34, and 66 days. The decrease in strength ranged from 20% after 7 days to 44% after 66 days [15]. The composition of the glass fibers determines their chemical resistance to alkaline environments. ARGFs have little boron, and they are enriched with zircon, which improves their chemical resistance compared with other types of glass fibers. The action of an alkaline environment (K_2_SiO_3_) ultimately causes glass fibers to dissolve or decompose. Elevated temperature accelerates the fiber degradation [10,11,19,21,23,28,29,30]. Previous studies [7,9,11,12,17] have described the formation of a corrosion shell on fiber surfaces in an alkaline environment. The shell consists of insoluble incrustations formed by the reaction between OH^-^ and oxides from the Si–O–Si chain of mineral fibers. Förster et al. partially described the growth phases of the corrosion shell and their direct correlation with the fluctuations in the strength of basalt fibers [21]. 

### 3.2. Weight Loss and Nominal Weight

**Figure 2:**
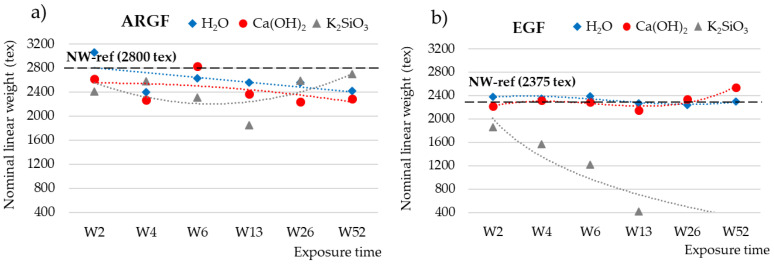
Nominal weights (tex) of (**a**) ARGFs and (**b**) EGFs treated in H_2_O, Ca(OH)_2_, and K_2_SiO_3_ according to the exposure time. The points represent the measured values for the nominal weight, and the lines represent the trend lines. The nominal weights of the reference samples were (**a**) 2800 tex for the ARGFs and (**b**) 2375 tex for the EGFs.

The weights of the untreated and treated fibers were used to calculate the nominal weight (tex), which is the weight (g) for a length of 1000 m of longitudinal fabric. Figure 2 shows how the nominal weight (NW) of the fibers fluctuated over time in different environments. For the ARGFs, the NW varied depending on the solution. The reference sample had an NW of 2800 tex (Figure 2a, dashed black line). In H_2_O, the organosilane coating of the ARGFs hydrolyzed, which caused the NW to increase in W2. Condensation and the elimination of water progressed in W4, which caused the NW to decrease slightly (blue line). In Ca(OH)_2_, the NW fluctuated during the exposure time up to W52 but showed an overall decreasing trend (red line). 

For the EGFs, the reference sample had an NW of 2375 tex (Figure 2b, dashed black line). In H_2_O, the NW oscillated around the reference value at W2–W52 (blue line). In Ca(OH)_2_, the NW was below the reference value at W2–W13 and then increased toward the end of the experiment at W26–W52 (red line). In K_2_SiO_3_, the NW showed a sharply declining trend, and the EGF completely dissolved after W13 (gray line).

### 3.3. Chemical Resistance

**Figure 3:**
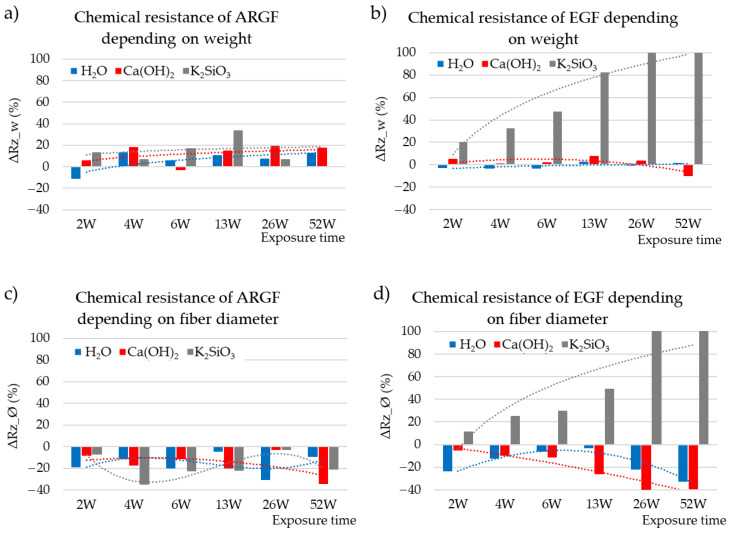
Chemical resistances of (**a**) ARGFs and (**b**) EGFs depending on weight (ΔRz_w (%)) and chemical resistances of (**c**) ARGFs and (**d**) EGFs depending on fiber diameter (ΔRz_Ø (%)).

The behavior of fibers in an alkaline environment can be predicted and modeled based on changes in the mechanical properties [7,9,10] or weight loss [11,12,25]. The change in weight is a significant parameter that determines the corrosion rate ΔRz, and it was calculated by using Equation (2) in this study. Figure 3a,b show the chemical resistances of ARGFs and EGFs over time (ΔRz_w calculation based on weight). The ARGFs lost some weight in all tested media: 6% in H_2_O, 12% in Ca(OH)_2_, and 15% in K_2_SiO_3_. Image analysis was used to measure the fiber diameter, and its chemical resistance (ΔRz_Ø, method in Equation (2) diameter instead of weight loss) over time is plotted in Figure 3c,d. Negative values in this graph indicate that the chemical resistance calculated based on fiber diameter increased. On average, the diameter of the ARGFs increased 15–18% in all environments.

The change in weight of EGFs was observed; the weight of the fibers in H_2_O remained constant. A slight decrease in chemical resistance was observed under the action of Ca(OH)_2_ solution within W6. No significant effect of the solution on the chemical resistance was observed until the end of the experiment (W52); the results even show a slight increase in the chemical resistance of the fibers. In K_2_SiO_3_, the fiber weight loss increased almost linearly and reached 100% in W26 and W52. The fiber diameter increased by an average of 16% in H_2_O. The fiber diameter increased linearly by 20% in Ca(OH)_2_. In contrast, the fiber diameter decreased in K_2_SiO_3_ by 30% at W2–W13, and the EGFs completely dissolved at W26–W52.

### 3.4. SEM Analysis

**Figure 4:**
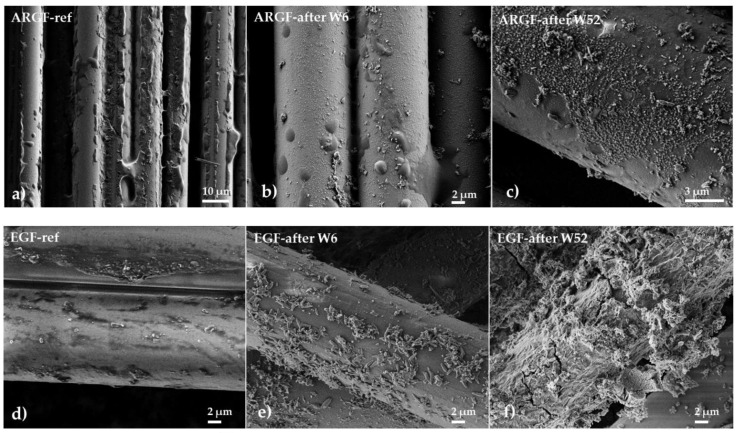
Surfaces of glass fibers after treatment in the Ca(OH)_2_ solution: ARGFs: (**a**) reference sample, (**b**) after W6, and (**c**) after W52; EGFs: (**d**) reference sample, (**e**) after W6, and (**f**) after W52.

Figure 4 shows the surfaces of the ARGFs during the experiment. ARGFs have an organosilane-based coating (sizing) to protect them from corrosion in an alkaline environment, which keeps them in the same shape for as long as possible. Figure 4b shows the ARGF in Ca(OH)_2_ after W6. The fiber surface is smooth and intact, whereas the sizing has formed disordered clusters, irregularly distributed over the fiber surface and in the inter-fiber space. Figure 4c shows the ARGF in Ca(OH)_2_ after W52. Calcium compounds formed by the fiber reacting with Ca(OH)_2_ have settled on the fiber surface, but there are no holes or cracks. Figure 4b shows the surfaces of EGFs in Ca(OH)_2_. Figure 4e shows the EGF in Ca(OH)_2_ in W6 when the first incrustations appeared on the fiber surface. Over time, the occurrence frequency of incrustations increased, and they gradually formed a continuous layer. Figure 4f shows EGF in Ca(OH)_2_ in W52, at which time the corrosion shell had broken and was slowly being torn off.

**Figure 5:**
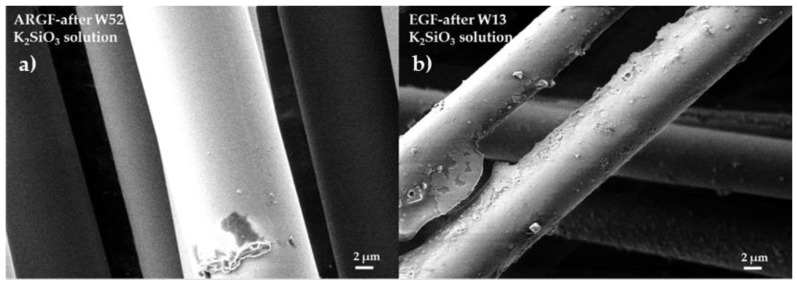
Surfaces of glass fibers treated with the K_2_SiO_3_ solution: (**a**) ARGF after W52 and (**b**) EGF after W13.

Figure 5a shows the surface of the ARGF in K_2_SiO_3_ at W52. The fiber surface is smooth without cracks and holes, and no partial or continuous incrustations can be observed. Figure 5b shows the surface of the EGF in K_2_SiO_3_ at W13 (the maximum time when the fibers were still observable). Incrustations of minor and more significant dimensions can be identified on the fiber surface. After W13, the EGFs completely dissolved. Thus, the EGFs had negligible resistance against an alkaline environment. 

The overall declining trend at a nominal weight, using a solution of Ca(OH)_2_ (Figure 2a, red line), can be attributed to the slow and sporadic increase in calcium incrustations on the fiber surface (Figure 4a–c. In K_2_SiO_3_, the NW increased at W2–W4, decreased at W6–W13, and then increased again from W26 onwards. The overall trend was an increase in NW over time (gray line). This may be attributed to the fibers adhering to each other to form complex and irregularly spaced formations (Figure 5a).

**Figure 6:**
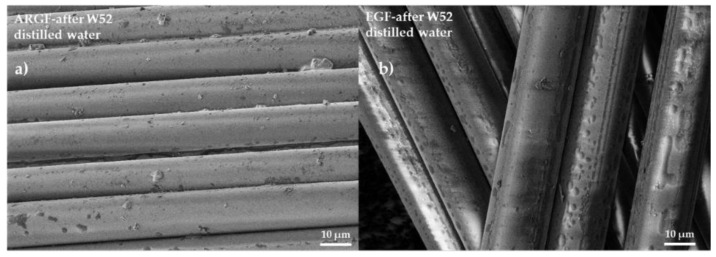
Surfaces of glass fibers treated with distilled water: (**a**) ARGF after W52, and (**b**) EGF after W52.

Figure 6a shows the surface of the ARGF in H_2_O at W52. The fiber surface is smooth with sizing residue, although the amount of residue is significantly less than in the reference sample (see Figure 4a). Figure 6b shows the surface of the EGF in H_2_O at W52. The fiber surface is identical in appearance to that of the reference sample (see Figure 4d).

### 3.5. Determination of Fiber Diameter by Image Analysis

**Figure 7:**
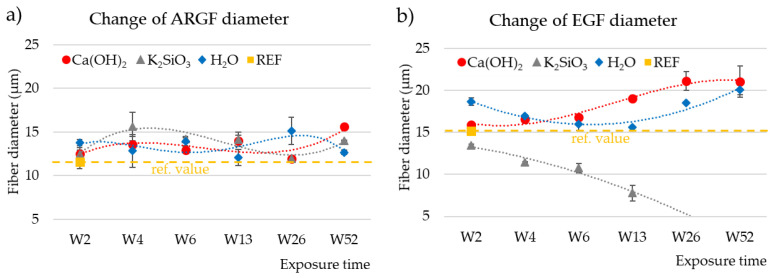
Changes in fiber diameter as a function of exposure time in different solutions: (**a**) ARGFs, and (**b**) EGFs. The points represent the average of measured values for the fiber diameter, and the lines represent the trend lines. The reference value for ARGFs fibers is 11.62 ± 0.84 µm and for EGFs fibers is 15.18 ± 0.17µm.

Figure 7a graphs the change in diameter of the ARGFs in H_2_O, Ca(OH)_2_, and K_2_SiO_3_. The reference samples had an average fiber diameter of 11.62 ± 0.8 µm. In both alkaline solutions (Ca(OH)_2_, K_2_SiO_3_), the fiber diameter increased by 1 µm at the beginning of the experiment (W2), and it increased by 2 µm in H_2_O (W2). The increase in diameter can be attributed to the action of water, which was present in all solutions. A high-silica layer formed on the fiber surface [20]. The diameter of the H_2_O-treated fibers oscillated around 12 µm for the rest of the experiment. The diameter of the Ca(OH)_2_-treated ARGF fibers increased to 13–15.64 µm during the experiment with a slowly increasing trend line. The K_2_SiO_3_-treated fibers reached a maximum fiber diameter of 15.65 ± 1.63 µm in W4, and the fiber diameter decreased during W6–W26 to 11.94 ± 0.03 µm before increasing to 14.02 ± 0.02 µm in W52. The trend line showed a slow decrease.

Figure 7b graphs the change in diameter of the EGFs in H_2_O, Ca(OH)_2_, and K_2_SiO_3_. The reference sample had an average diameter of 15.18 ± 0.17 µm. The diameter of the H_2_O-treated fiber remained greater than that of the reference sample throughout the experiment. The water increased the EGF fiber diameter, which oscillated around 17 µm. The diameter of the Ca(OH)_2_-treated EGF increased to 15.91 ± 0.29 µm in W2 and 21.07 ± 1.88 µm in W52. The trend line was growing. The diameter of the K_2_SiO_3_-treated EGF showed a significant decreasing trend. The fiber diameter decreased from the beginning of the experiment to a final value of 7.7 ± 0.93 µm in W13. The fiber dissolved after further exposure, therefore no further data could be obtained for W26–W52.

### 3.6. Statistical Analysis

#### 3.6.1. Trends or Patterns over Time

**Figure 8:**
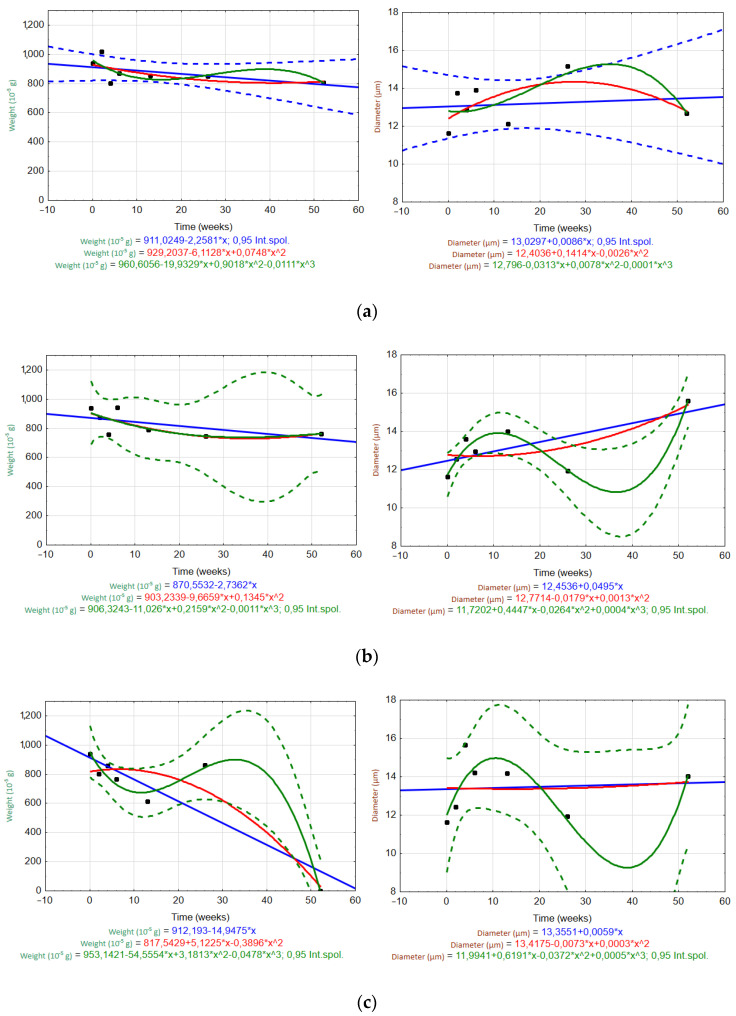
Scatter plots of the temporal trends for the ARGF weight (left) and fiber diameter (right) in different solutions: (**a**) H_2_O, (**b**) Ca(OH)_2_, and (**c**) K_2_SiO_3_ (Int.spol. mean confidence interval).

**Figure 9:**
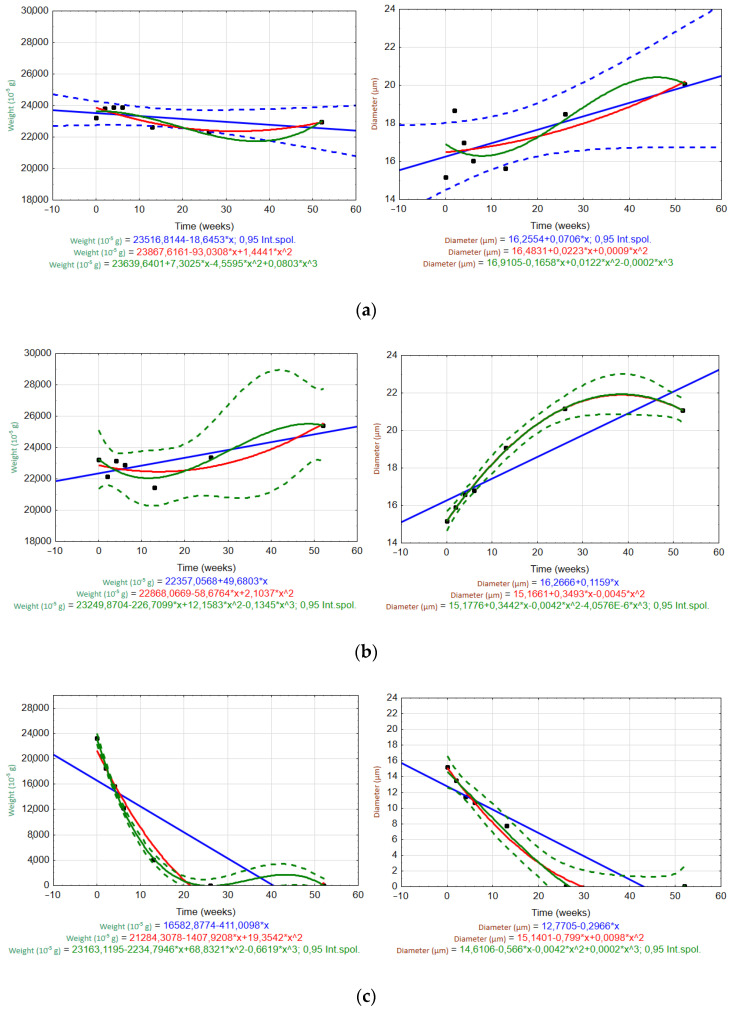
Scatter plots of the temporal trends for the EGF weight (left) and fiber diameter (right) in different solutions: (**a**) H_2_O, (**b**) Ca(OH)_2_, and (**c**) K_2_SiO_3_. (Int.spol. mean confidence interval).

Figure 8 and Figure 9 show scatter plots used to evaluate the trends of the fiber weight and diameter over time (W0–W52) in different solutions (H_2_O, Ca(OH)_2_, K_2_SiO_3_). Measured data are plotted as black dots, and the interpolated trends are represented by blue (linear), red (quadratic), and green (cubic) lines. The regression equations are listed in the graphs. Regression bands are shown as dashed lines with the same corresponding color at a confidence level of 0.95. For clarity, only one confidence interval is displayed (the one for the most appropriate trend).

For the ARGF in H_2_O (Figure 8a), only the linear trend accurately predicted the changes in the fiber weight and diameter over time (rate of weight loss was 0.32 × 10^−5^ g/day; rate of diameter increase was 0.0014 µm/day). For the ARGF in Ca(OH)_2_ (Figure 8b), the quadratic and cubic trends accurately predicted the change in fiber weight over time. However, only the cubic trend was suitable for predicting the fiber diameter. The same result was observed for the ARGF in K_2_SiO_3_ (Figure 8c).

For the EGF in H_2_O (Figure 9a), only the linear trend (blue line) may predict the changes in the fiber weight and diameter over time (rate of weight loss was 2.57 × 10^−5^ g/day; rate of diameter increase was 0.01 µm/day). These results show that the ARGFs lost weight at a rate about eight times slower than EGFs, and the diameter increased at a rate about seven times slower than EGFs. The EGF in Ca(OH)_2_ (Figure 9b) showed that both the quadratic and cubic trends were suitable for predicting the changes in weight and diameter over time. The same results were observed for the EGF in K_2_SiO_3_ (Figure 9c). However, the weight loss over time could also be predicted by using a linear trend (i.e., it corresponded to the change in diameter).

#### 3.6.2. Relationships of Variables

**Figure 10:**
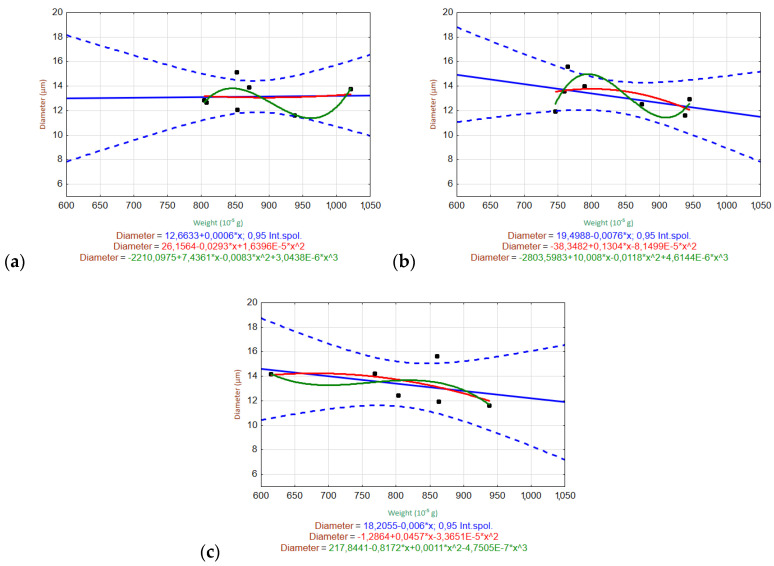
Scatter plots of the relationship between the fiber diameter and weight of ARGFs treated with different solutions: (**a**) H_2_O, (**b**) Ca(OH)_2_, and (**c**) K_2_SiO_3_ (bottom).

**Figure 11:**
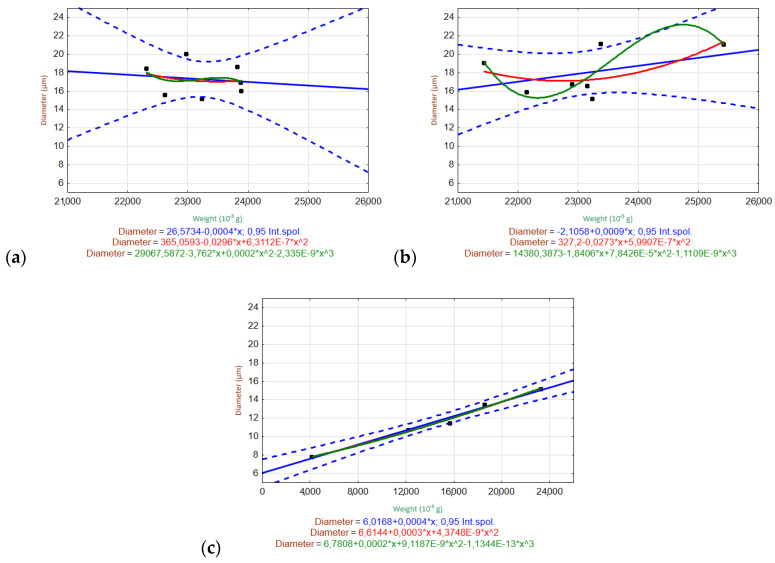
Scatter plots of the relationship between the fiber diameter and weight of EGFs treated with different solutions: (**a**) H_2_O, (**b**) Ca(OH)_2_, and (**c**) K_2_SiO_3_ (bottom).

Figure 10 and Figure 11 show the scatter plots used to evaluate the relationships between the weight and fiber diameter within the given measurement ranges for the tested solutions (H_2_O, Ca(OH)_2_, K_2_SiO_3_). The measured data are plotted as black dots, and the interpolated relationships are shown as blue (linear), red (quadratic), and green (cubic) lines. The regression equations are listed in the graphs. The regression bands are shown as dashed lines with the same corresponding color at a confidence level of 0.95. For clarity, only one confidence interval is displayed (the one for the most appropriate trend).

The ARGF in H_2_O (Figure 10) did not show any relationship between the fiber diameter and weight. This indicates that the fiber diameter did not change significantly with the decrease in weight during the experiment (up to W52). The ARGF in Ca(OH)_2_ showed that the linear and quadratic trends accurately predicted the relationship between the fiber weight and diameter, although the linear trend showed slightly better statistical significance (indicated by the narrower confidence interval). The same result was observed for the ARGF in K_2_SiO_3_.

The EGF in H_2_O (Figure 11) generally showed no relationship between the fiber diameter and weight. The fiber diameter showed a very moderate increase, with a decrease in weight during the experiment (up to W52). The EGF in Ca(OH)_2_ showed that the linear and quadratic trends could be used to predict the relationship between the fiber weight and diameter, although the linear trend showed slightly better statistical significance owing to the narrower confidence interval. The EGF in K_2_SiO_3_ showed a linear relationship between the fiber diameter and weight, where the diameter decreased linearly with decreasing weight.

#### 3.6.3. Dependencies of Three Variables

**Figure 12:**
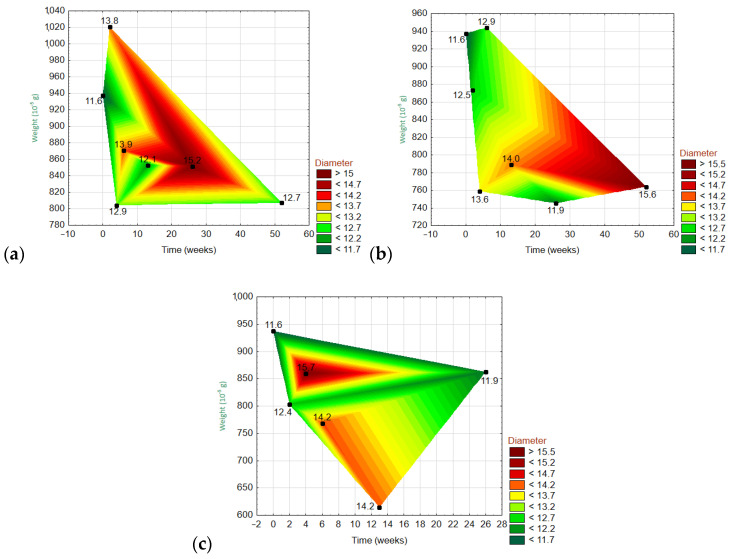
Three-dimensional wafer plots showing the dependencies of the three variables (diameter, weight, and time) for ARGFs treated with different solutions: (**a**) H_2_O, (**b**) Ca(OH)_2_, and (**c**) K_2_SiO_3_ (bottom).

**Figure 13:**
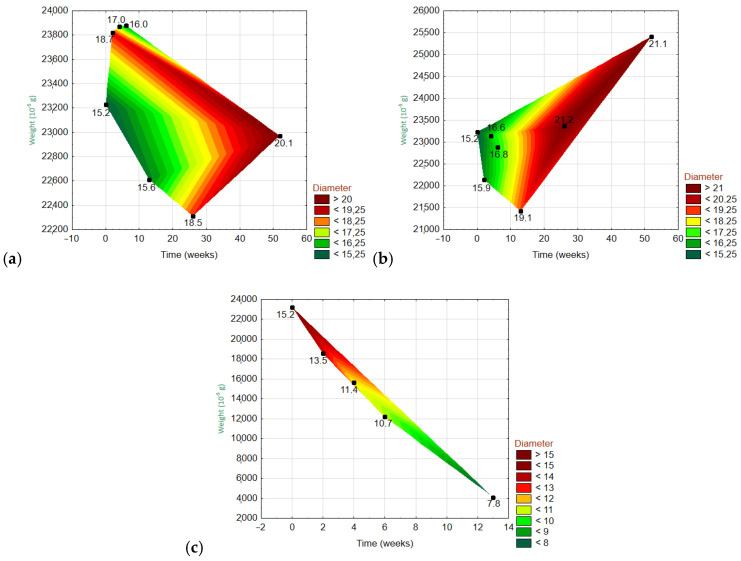
Three-dimensional wafer plots showing the dependencies of the three variables (diameter, weight, and time) for EGFs treated with different solutions: (**a**) H_2_O, Ca(OH)_2_, and (**c**) K_2_SiO_3_ (bottom).

Figure 12 and Figure 13 show the 3D wafer plots of the dependencies of the three variables: the fiber weight (y-axis), fiber diameter (z-axis), and exposure time (x-axis). On the z-axis, the lowest values are in dark green, and the highest values are in dark red. The exact values are given on the right side of each graph. For the ARGF in H_2_O (Figure 12), the weight fluctuated over time with an overall declining trend (i.e., the right corner of the polygon points downwards). Meanwhile, the fiber diameter was greatest at W20–W30 and then gradually decreased. For the ARGF in Ca(OH)_2_, the fiber weight again fluctuated with time with an overall declining trend. The fiber diameter gradually increased throughout the experiment. For the ARGF in K_2_SiO_3_, the fiber weight remained stable over time. Meanwhile, the fiber diameter was greatest at W4–W10 and then dramatically decreased later.

For the EGF in H_2_O (Figure 13), the fiber weight fluctuated over time with an overall declining trend. Meanwhile, the fiber diameter increased over time, and it did not show a relationship with the weight. For the EGF in Ca(OH)_2_, the fiber weight fluctuated over time with an overall increasing trend (i.e., the right corner of the polygon points upwards). The fiber diameter increased over time, and it showed no relationship with the weight. For the EGF in K_2_SiO_3_, the fiber weight decreased dramatically over time (from start to 13W). The fiber diameter gradually decreased during this same period.

The H_2_O-treated ARGFs showed minimal weight loss. The tensile strength increased because of the increased fiber diameter. The evaluation of the chemical resistance confirmed minimal weight loss and increased fiber diameter. The Ca(OH)_2_-treated ARGFs showed a decrease in weight and an increase in fiber diameter, which were accompanied by a decrease in tensile strength. The Ca(OH)_2_ solution dissolved the protective coating and caused the formation of small and dispersed incrustations on the fiber surface. The K_2_SiO_3_-treated ARGFs showed an increase in weight and a decrease in fiber diameter, which were accompanied by a decrease in tensile strength. SEM analysis did not observe an increase in insoluble incrustations on the fiber surface, which remained smooth and clean.

The H_2_O-treated EGFs did not show weight loss. The tensile strength decreased, even though the fiber diameter increased slowly. The Ca(OH)_2_-treated EGFs showed a slow increase in weight. The fiber diameter increased, but the tensile strength decreased. This is because the Ca(OH)_2_ solution reacted with the glass fiber, which precipitated insoluble incrustations onto the fiber surface that merged into an integrated coating. The K_2_SiO_3_-treated EGFs showed decreases in the fiber weight, diameter, and tensile strength. SEM analysis observed the growth of insoluble incrustations on the fiber surface, and the fibers dissolved at W13. The degradations of ARGFs treated with Ca(OH)_2_ or K_2_SiO_3_ over time could be predicted by cubic trends for the fiber weight and diameter. Very similar results were observed for the EGFs in both alkaline solutions. The ARGFs and EGFs showed linear relationships between the fiber weight and diameter in Ca(OH)_2_. A similar result was observed for ARGFs in K_2_SiO_3_. Meanwhile, the EGFs in K_2_SiO_3_ showed a strong linear relationship between the fiber diameter and weight.

## 4. Conclusions

The tensile strength of water-treated ARGF fibers increases (by 32%) due to the increase in fibers diameter. The weight of Ca(OH)_2_-treated ARGFs shows a decrease, and the fiber diameter increases; the tensile strength of the fibers reduces (by 50%) compared to the reference value. The weight of K_2_SiO_3_-treated ARGFs increases, and the fiber tensile strength decreases (by 85%) due to the reduction in fiber diameter. SEM analysis did not show any increase in the insoluble incrustations on the surface of the fibers, and the surface of the fibers is smooth and clean.

EGFs in water do not show weight loss; the tensile strength decreases (up to 68%), although the fiber diameter increases slowly. The weight Ca(OH)_2_ treated EGF slowly increases. The weight, fiber diameter, and tensile strength (up to 95%) of EGF decreases after treatment with K_2_SiO_3_. SEM analysis proved a growing insoluble incrustation on the fiber surface. The fibers are dissolved in 13 weeks.

A method was developed for the nondestructive testing of fiber resistance based on image analysis. In this study, correlations were obtained between image analysis and conventional methods based on changes in fiber weight or tensile strength to validate the developed method. Analyzing the changes in fiber properties introduces some errors in the form of probability. The developed method based on image analysis complements current evaluation methods by helping visualize the ongoing changes to the fiber surface (e.g., incrustations or corrosion shells) due to the action of alkaline and neutral environments.

## Data Availability

The data presented in this study are available on request from the corresponding author.
